# World’s first en bloc heart-lung transplantation using the paragonix lungguard donor preservation system

**DOI:** 10.1186/s13019-023-02281-7

**Published:** 2023-04-11

**Authors:** Daniel Neto, Brandon Guenthart, Yasuhiro Shudo, Maria E. Currie

**Affiliations:** Department of Cardiothoracic Surgery, Center for Academic Medicine, Stanford University School of Medicine, 453 Quarry Road, Stanford, CA 94305 USA

**Keywords:** Biotechnology, Cardiac transplant, Pulmonary hypertension

## Abstract

We present the first *en bloc* heart-lung donor transplant procurement using the Paragonix LUNGguard™ donor preservation system. This system offers reliable static hypothermic conditions designed to prevent major complications such as cold ischemic injury, uneven cooling and physical damage. While this represents a single case, the encouraging results warrant further investigation.

## Background

Organ transplantation is a reliable and effective method to treat end stage organ failure. Around 150,000 solid organs are successfully transplanted yearly across the globe with heart and lung 5-year survival rate exceeding 50% [1]. Highly standardized protocols and techniques are employed during solid organ procurement preservation and transportation to protect donor allografts and enable the utilization of more organs. Over the last several decades, a myriad of advances in both heart and lung preservation systems has led to innovative solutions to meet the growing demand and expand the donor pool of organs. For example, in 1987 Hardesty and Griffith reported an autoperfusion technique to improve preservation of donor heart-lungs [2], but the method was quite complex, therefore most cases performed continue using donor organs preserved on ice. Given their relative scarcity, little has been done to address optimal storage and transport conditions for heart-lung *en bloc* grafts.

We present the first case using the Paragonix LUNGguard™ System for storage and transportation of a heart-lung *en bloc* donor allograft. This system offers reliable static hypothermic conditions designed to prevent major complications such as cold ischemic injury, uneven cooling and physical damage.

## Case presentation

**Fig. 1:**
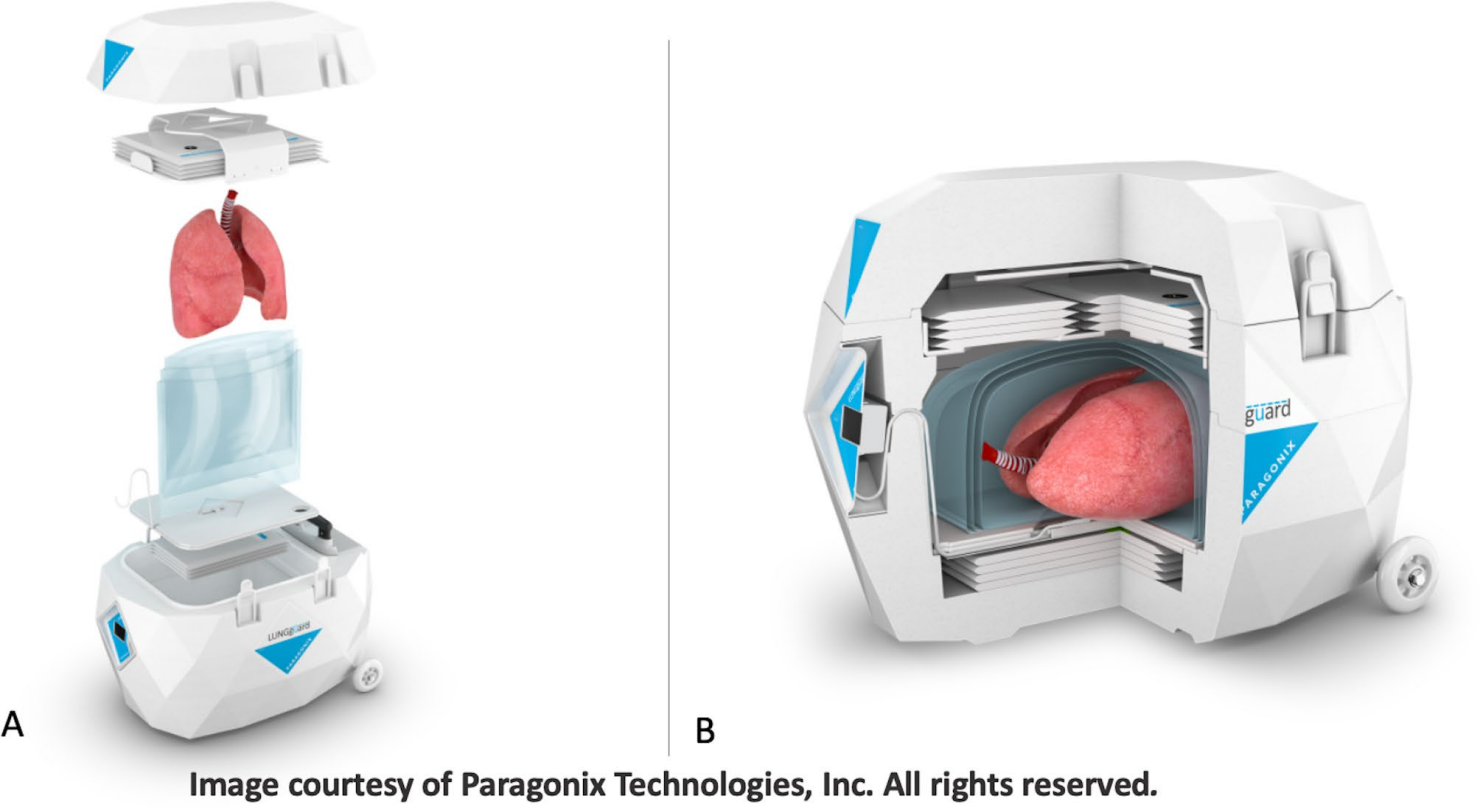
LUNGguard™ device components (A) and closed with allograft in place (B). The device components from top to bottom are: the shipper lid, the SherpaCool™ Tray, the allograft, the sterile isolation bags, the bottom tray of the device, another SherpaCool™ Tray

**Fig. 2:**
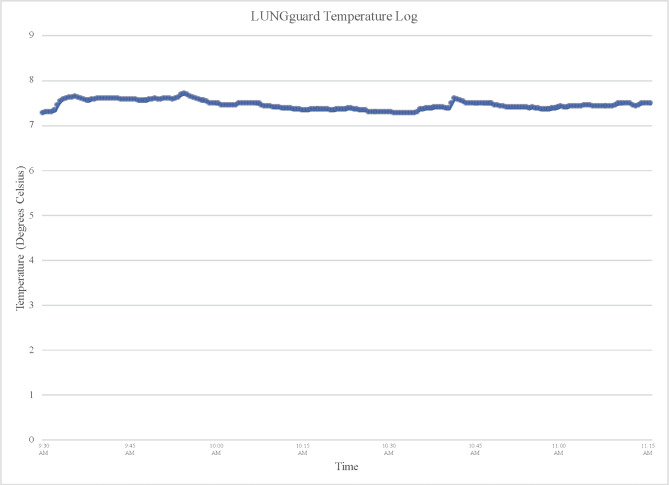
LUNGguard™ temperature log during transport of heart-lung allograft from donor hospital to recipient operating room. Temperature accuracy is ± 0.5 °C from 0° to 50 °C. Logged temperature data from the LUNGguard™ system can be accessed through the Paragonix mobile application

A 35-year-old male with scleroderma associated interstitial lung disease, pulmonary arterial hypertension, right ventricular dysfunction, esophageal dysmotility, and Raynaud’s was listed for heart-lung transplantation. The patient was admitted to the hospital for acute on chronic respiratory failure. A suitable brain-dead donor was identified. The donor was a 14-year-old female whose cause of death was anoxia due to suspected drug intoxication. The donor had an acceptable size match to the recipient, normal biventricular function (EF 56%), clear chest imaging and adequate oxygenation (Pa0_2_ 382 mm Hg). The heart-lung bloc was procured from a hospital located 1.5 hours away using a standard procurement technique [3]. Specifically, 1L of University of Wisconsin solution was administered to the heart and 3 L of Perfadex® was administered to the lungs without a retrograde flush. The heart-lung allograft was in 4 L of PhysioSol^TM^ preservation solution at 4°C within sterile isolation bags. The sterile bag was de-aired with the heart-lung allograft suspended in preservation solution then then secured and packaged using the LUNGguard™ Transport System (Fig. 1). The total storage period for the allograft was 172 minutes. The LUNGguard™ Transport System measured and recorded temperatures during allograft transport (Fig. 2). The average probe temperature measured by the LUNGguard system was 7.42°C and the average ambient temperature was 17.34°C.

**Fig. 3:**
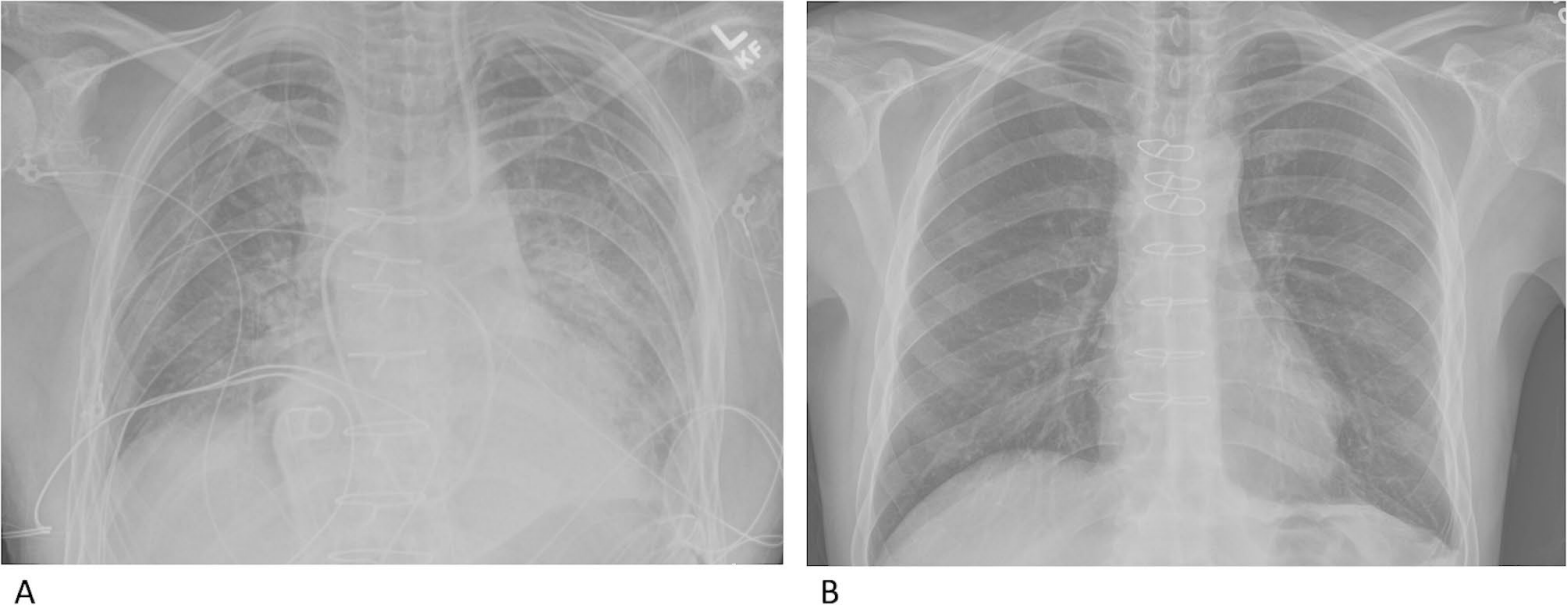
Postoperative chest xray at postoperative day two (A) and three months following heart-lung transplant (B)

The recipient underwent median sternotomy. Cardiopulmonary bypass was initiated at 35 °C with aortic and bi-caval cannulation. Explant and implant procedures of the heart-lung bloc were performed in standard fashion [4]. The allograft ischemic time was 208 min, including a warm ischemic time of 36 min. The recipient cardiopulmonary bypass time and aortic cross clamp time were 169 and 115 min, respectively. The patient recovered well, with no major complications. No mechanical circulatory support was required. The patient was extubated on postoperative day one with a P/F ratio of 367. The patient was discharged on postoperative day 16. At one year post-transplant, the patient is recovering at home and continues to have excellent function with no allograft vasculopathy (Fig. 3). An echocardiogram completed one year post transplant revealed biventricular function was normal by echocardiographic assessment (LVEF 62%).

## Discussion and conclusions

Dr Bruce Reitz was the pioneer in heart-lung transplantation, and the first successful procedure was at Stanford Hospital in 1981 [4]. Since then, over 4,000 similar procedures for end-stage heart and lung disease were done around the world [5]. Our institution have a 100% 30-day and 1-year survival following en bloc heart-lung transplant [6].

Technology and techniques related to transplantation have evolved to meet the healthcare demands required in the 21st century. Among the most important and challenging are those related to preservation and transport of solid organs. Recently the development of new technologies in preservation has become an increasing priority in United States, [7–9] including the utilization of marginal donors, use of ex-vivo perfusion and acceptance of organs after circulatory death (DCD), in an effort to expand the organ donor pool [10].

Temperature is a cornerstone for the solid organ preservation along with the flush with a specialized preservation solution and storage in crystalloid solution. Cold storage systems are used to keep the temperature low to reduce cellular and metabolic demand [11–13]. Lower organ temperatures (0 to 4 °C) maintain high energy phosphates [12–15]. Cold static preservation, that is, preservation on ice, remains the clinical standard for short-term organ preservation due to its simplicity and low cost [16]. However, temperatures below 2 °C significantly increase the risk of cold injury with some proteins denaturing below 0 °C [12–15]. Storage on ice may result in uneven organ cooling and freeze injury, mainly during prolonged storage, when the temperature inside the cooler can reach below 0 °C [17]. Freeze injury restricts tissue survival by contributing to structural damage that prevents revascularization and compromises both the attachment and integrity of the tissues [1, 16]. When freeze injuries are associated with prolonged ischemic times (approximately 6 h for hearts and 8 h for lungs), these injuries may contribute to Primary Graft Dysfunction (PGD). PGD phenomenon related to ischemia-reperfusion injury that regularly occurs during the cold ischemic time [18], with high associated early and late mortality rates in heart and lung transplantation [19]. In this case, the ischemic time was limited to 3 h and 28 min; therefore, at low risk of allograft injury.

Between 8 and 12 °C, organ function is maintained to a greater extent [20, 21]. However, at temperatures above 12 °C higher metabolic demand may lead to irreversible hypoxic injury and impairment of organ function. Based on these findings, 4 − 8 °C has been proposed as the ideal temperature for organ preservation [11].

The Paragonix LUNGguard™ Donor Lung Preservation System is a preservation device designed and used for donor lungs, and an analogous device, the Paragonix SherpaPak, has been used for donor hearts. These devices provide a controlled storage temperature during transportation and avoid direct allograft to the ice contact. The device also monitors and transmits live temperature data via Bluetooth linked to a cell phone application. In our case temperature was maintained between 6.84 and 7.69 °C. As demonstrated in this case, the novel application of the LUNGguard™ system with a heart-lung *en bloc* allograft resulted in excellent graft function and favorable post-transplantation outcome. However, further trials comparing the LUNGguard™ system to other cold preservation techniques for *en bloc* heart-lung allografts are required.

Currently no device has been designed to meet the unique needs of heart-lung *en bloc* allografts. As new organ transplant policy changes have expanded the donor pool to further distances, organ preservation systems and protocols must also consider heart-lung *en bloc* allografts. Here we describe the novel application of the LUNGguard™ storage system to provide safe and reliable static hypothermic storage and transport conditions in case of heart-lung *en bloc* transplantation. While this represents a single case, the encouraging results warrant further investigation.

## Data Availability

Not applicable to this study.
